# The specific distribution pattern of *IKZF1* mutation in acute myeloid leukemia

**DOI:** 10.1186/s13045-020-00972-5

**Published:** 2020-10-20

**Authors:** Xiang Zhang, Xuewu Zhang, Xia Li, Yunfei Lv, Yanan Zhu, Jinghan Wang, Jie Jin, Wenjuan Yu

**Affiliations:** 1 Department of Hematology, The First Affiliated Hospital, Zhejiang University School of Medicine, #79 Qingchun Rd Zhejiang Province, Hangzhou, 310003 China; 2 Key Laboratory of Hematologic Malignancies, Diagnosis and Treatment, Zhejiang, Hangzhou, Zhejiang China

**Keywords:** *IKZF1* mutation, Acute myeloid leukemia, Recurrence

## Abstract

IKZF1 belongs to the IKAROS family of transcription factors, and its deletion/mutation frequently affects acute lymphoblastic leukemia. In acute myeloid leukemia, *IKZF1* deletion has been demonstrated recurrent, but whether *IKZF1* mutation also exists in AML remained largely unknown. Herein, we analyzed the *IKZF1* mutation in AML. In our cohort, the frequency of *IKZF1* mutation was 2.6% (5/193), and 5 frameshift/nonsense mutations as well as 2 missense mutations were identified in total. Molecularly, *IKZF1* mutation was absent in fusion gene-positive AML, but it was demonstrated as the significant concomitant genetic alteration with *SF3B1* or *bi-allele* *CEBPA* mutation in AML. Clinically, two *IKZF1, PTPN11* and *SF3B1-*mutated AML patients exhibited one aggressive clinical course and showed primary resistant to chemotherapy. Furthermore, we confirmed the recurrent *IKZF1* mutation in AML with cBioPortal tool from OHSU, TCGA and TARGET studies. Interestingly, OHSU study also showed that *SF3B1* mutation was the significant concomitant genetic alteration with *IKZF1* mutation, indicating their strong synergy in leukemogenesis. In conclusion, *IKZF1* mutation recurrently affected AML.

IKZF1 belongs to the IKAROS family of transcription factors. It contains four zinc fingers at the N-terminal that directly bind to DNA at the core motif A/GGAAA and additional two zinc fingers at the C-terminal required for forming homo- and hetero-dimerization between different IKZF proteins [1]. DNA binding activity of IKZF1 can be enhanced by its dimerization, so both DNA-binding and dimer-forming defects alter IKZF1 function. *IKZF1* deletions and mutations have been reported to affect B-cell precursor ALL and contribute to its poor prognosis [2]. *IKZF1* alterations are less studied in AML. Recurrent *IKZF1* deletions have been identified in AML [3, 4], but whether *IKZF1* mutations affect AML in general remains unknown. Herein, we analyzed *IKZF1* mutation in AML.

A total of 193 adult AML patients, who subjected to TES, were retrospectively analyzed in our center (01/05/2018–29/02/2020), while APL was excluded. Among these patients, 100 were male and 93 were female, and the median age was 56 (range 18–82). A total of 169 patients were diagnosed with *de novo* AML, 10 with refractory/relapsed AML, 6 with MDS/AML, 5 with MLL (5 de novo cases), and 3 with MS/AML (1 de novo case, 2 refractory/relapsed cases). The panel of TES included 236 genes recurrently mutated in hematological malignancies, and TES was displayed by NovaSeq platform (Illumina). The average raw sequencing depth on target per sample was ≥ 1000, and VAF ≥ 1% was considered significant. For TES, 184 samples were collected from BM and 9 samples from PB. In addition, fusion gene screening for common rearrangements in AML was employed.

*IKZF1* mutation affected 2.6% (5/193) or 1.8% (3/169) of all AML patients or *de novo* AML patients from our cohort, respectively (Fig. 1a and Table 1). Totally, 7 different types of *IKZF1* mutations were found, and 5 were frameshift or nonsense mutations, while 2 were missense mutations (Fig. 1b). Interestingly, *IKZF1* mutation was absent in fusion gene-positive AML, while *IKZF1* mutation co-occurred with *PTPN11*, *SF3B1*, *bi-allelic* *CEBPA* or *WT1* mutation in our study (Fig. 1c). Their association was further determined by Chi-square test with continuity correction, and OR was calculated. In 154 fusion gene-negative patients, we found that *SF3B1* and *bi-allelic* *CEBPA* but not *PTPN11* or *WT1* mutations were the significant concomitant genetic alteration with *IKZF1* mutation (*P* < 0.05; OR > 1) (Fig. 1d–f). In clinic, treatment response was evaluated in 4/5 patients with *IKZF1*-mutated AML, and CR was achieved in 2 patients. Notably, 2 primary chemotherapy-resistant patients had *IKZF1, PTPN11* and *SF3B1-*mutated AML, so this subtype of AML seemingly exhibited an aggressive clinical course. However, the impact of *IKZF1* mutation in AML could not be determined in our study due to limited positive cases and short follow-up duration.

**Fig. 1:**
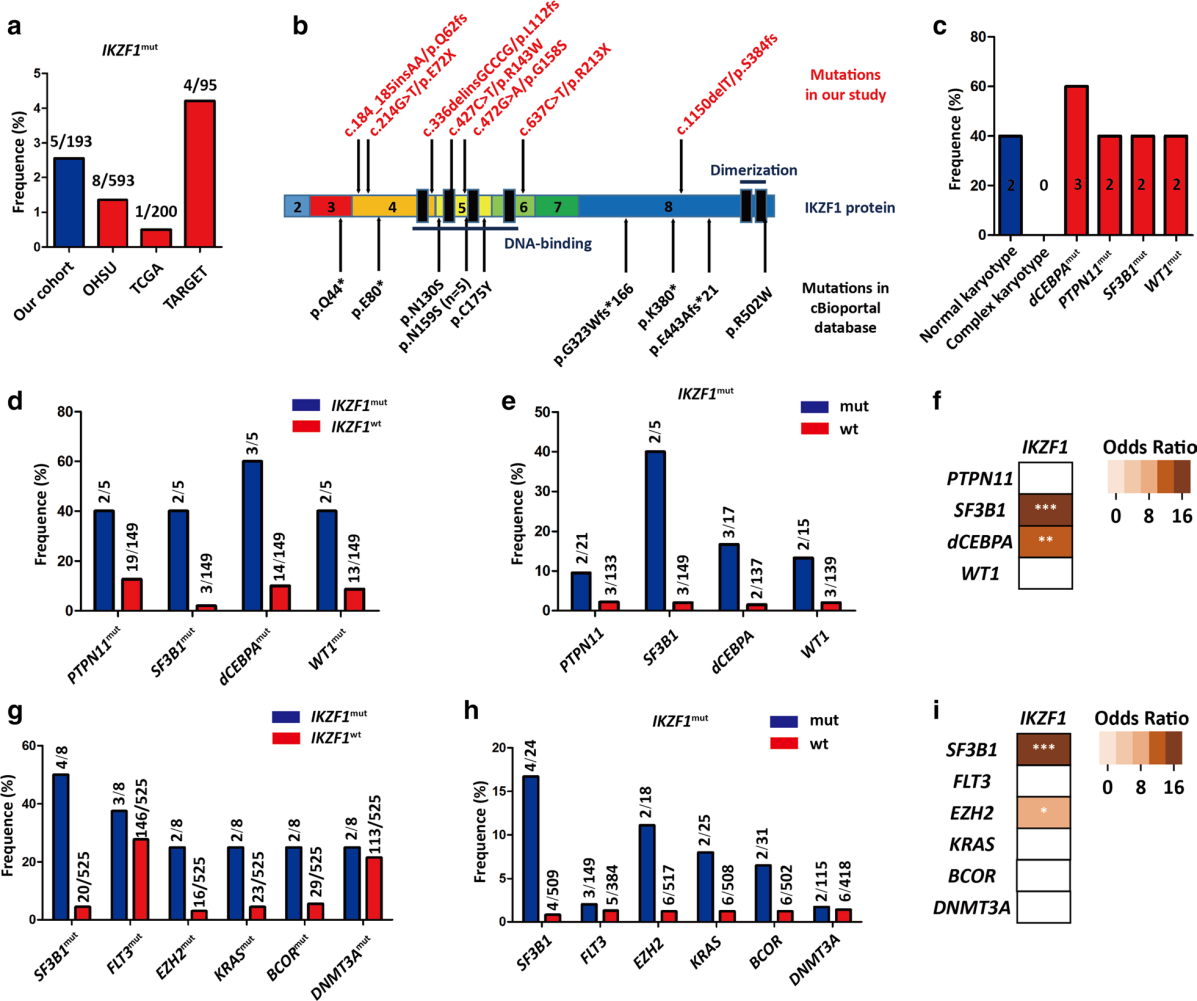
*IKZF1* mutation in AML. **a** The frequency of *IKZF1* mutation in our AML cohort and literature reports. **b** The type of *IKZF1* mutation identified in our cohort and literature reports. **c** Genetic lesion in AML with *IKZF1* mutation. **d** The frequency of *PTPN11*, *SF3B1*, *bi-allele CEBPA* and *WT1* mutations in *IKZF1*^mut^-AML and *IKZF1*^wt^-AML from our cohort, respectively. **e** The frequency of *IKZF1* mutation in *PTPN11*, *SF3B1*, *bi-allele CEBPA*, *WT1*-wild type/-mutated AML from our cohort, respectively. **f** The statistical significance of associations between *IKZF1* and other gene mutations in our study was assessed by Chi-square test with continuity correction. Odds ratio was also calculated to define whether the correlation was positive or negative. **g** The frequency of *SF3B1*, *FLT3*, *EZH2*, *KRAS*, *BCOR* and *DNMT3A* mutations in *IKZF1*^mut^-AML and *IKZF1*^wt^-AML from OHSU study, respectively. **h** The frequency of *IKZF1* mutation in *SF3B1*, *FLT3*, *EZH2*, *KRAS*, *BCOR* and *DNMT3A*-wild type/-mutated AML from OHSU study, respectively. **i** Associations between *IKZF1* and other gene mutations in OHSU study were also analyzed as **f** indicated. NS, *P* ≥ 0.05; *, *P* < 0.05; **, *P* < 0.01; ***, *P* < 0.001

**Table 1:** Acute myeloid leukemia with *IKZF1* mutation in our cohort

No.	Gender/age	Diagnosis	PB	BM blast	*IKZF1* mutation (VAF, mutational site)	Karyotype	Gene fusion	Gene mutation	Response	OS
1	M/45	AML-M0	WBC 19.1 G/L HB 77 g/l PLT 129 G/L Blast 89%	90%	44.51%, Exon4:c.184_185insAA/p.Q62fs*32	46,XY,t(3;3)(q13;q27)[10]	ND	*PTPN11,SF3B1*	HAA, NR	Dead, 3.5 months
2	M/61	AML-M0	WBC 5.2 G/L HB 83 g/l PLT 917 G/L	28%	38.19%, Exon4:c.214G>T/p.E72X 42.29%, Exon8:c.1150delT/p.S384fs*31	47,XY,+3(q21)[20]	ND	*BCOR,PTPN11,FLT3, SF3B1*	AZA + IDA, NR	Dead, 3 months
3	M/24	AML-M2	WBC 6.6 G/L HB 85 g/l PLT 10 G/L Blast 34%	57%	1.69%, Exon5:c.427C>T/p.R143W 3.49%, Exon6:c.637C>T/p.R213X	46,XY,del(8)(q22)[5]/46,XY[5]	ND	*dCEBPA,MSH6,DNMT3A,WT1*	IA, CR	Live, 5 months
4	M/21	MLL-M2	WBC 46.6 G/L HB 76 g/l PLT 23 G/L Blast 75%	88.5%	23.04%, Exon5:c.472G>A/p.G158S	46,XY[20]	ND	*CCND3,dCEBPA,GATA2*	VEN + CAG, CR	Live, 4 months
5	F/52	AML-M2/ MS (r/r)	WBC 4.2 G/L HB 98 g/l PLT 84 G/L	35%	19.93%, Exon4:c.336delinsGCCCG/ p.L112fs*4	46,XX[20]	ND	*dCEBPA,CSF3R,CTCF,WT1*	GHAA, NA	Live, 0.5 months

*PB* peripheral blood, *BM* bone marrow, *VAF* variant allele frequency, *OS* overall survival, *M* male, *F* female, *AML* acute myeloid leukemia, *MLL* mixed lineage leukemia, *AML/MS* acute myeloid leukemia with myeloid sarcoma, *R/R* relapsed or refractory, *WBC* white blood cell, *HB* hemoglobin, *PLT* platelet, *ND* not detected, *NR* no response, *CR* complete remission, *NA* not available

In addition to our study, we also used the cBioPortal tool to analyze the frequency of *IKZF1* mutation in other three independent studies (OHSU [5], TCGA [6] and TARGET [7]). The frequency was 1.35% (8/593), 0.5% (1/200) and 4.21% (4/95), respectively, while the relatively high frequency in our study was possibly attributed to the criterion of enrollment and the limited cases (Fig. 1a). In total, 13 mutations were found in these studies, but there were no patients with 2 different mutations simultaneously (Additional file 1: Table S1). Of these 13 patients, 6 had frameshift or nonsense mutations and the rest 7 had missense mutations. *IKZF1*^N195S^ was a hotspot mutation with the frequency of 38.5% (5/13), but it was absent in COSMIC and our study (Fig. 1b). Due to limited positive cases in TCGA and solely pediatric cases in TARGET, we further analyzed the related genetic events of *IKZF1* mutations in OHSU and found that *SF3B1* and *EZH2*, but not *KRAS*, *BCOR*, *FLT3* or *DNMT3A* mutations were the significant concomitant alteration with *IKZF1* mutation (Fig. 1g–i). Remarkably, *SF3B1* mutation appeared in both concomitant alteration lists of our study and OHSU, suggesting their strong synergy in leukemogenesis.

Compared to AML, *IKZF1* alteration is well studied in ALL. Churchman et al. reported that *IKZF1* alteration affected 25% of childhood and 44% of young adult pre-B-cell ALL, especially *BCR-ABL1*-positive ALL with frequency of over 80%. In ALL, the most common type of alterations in *IKZF1* is deletions, whereas *IKZF1* mutations accounted only 2.6% of childhood and 3.4% of young adult ALL. The latter were observed in 11.9% of *BCR-ABL1*-negative and 2.2% of *BCR-ABL1*-positive ALL cases [8]. The frameshift or nonsense mutations of *IKZF1* often occurred at the N-terminal or the region between DNA binding and dimerization domains, while missense mutations affected both domains. Consistently, *IKZF1* mutations followed the same pattern in AML. Similarly to *IKZF1*^N159S^ in AML, *IKZF1*^N159Y^ is a hotspot mutation in ALL that affects its DNA binding domain. *IKZF1*^N159Y^-ALL exhibited one unique transcriptional profile characterized by downregulation of B-cell receptor and JAK-STAT signaling and upregulation of *SALL1* [9]. Nevertheless, whether *IKZF1*^N159S^-AML could be defined as one independent subtype remains to be investigated.

In conclusion, besides of *IKZF1* deletion, *IKZF1* mutation is also recurrent in AML.

## Supplementary information

**Additional file 1: Table S1.** The variant allele frequency of *IKZF1* mutation in AML from cBioPortal database.

## Data Availability

All data generated or analyzed during this study are included in this published article.

## Abbreviations

ALL: Acute lymphoblastic leukemia
AML: Acute myeloid leukemia
APL: Acute promyelocytic leukemia
BM: Bone marrow
CR: Complete remission
MDS/AML: Myelodysplastic syndrome-transformed AML
MLL: Mixed lineage leukemia
MS/AML: Myeloid sarcoma with bone marrow infiltration
OR: Odds ratio
PB: Peripheral blood
TES: Targeted exome sequencing
VAF: Variant allele frequency
