# Supplementary material for: The specific distribution pattern of IKZF1 mutation in acute myeloid leukemia
Source: J Hematol Oncol. 2020 Oct 20;13:140. doi: 10.1186/s13045-020-00972-5 (PMC7574539; doi:10.1186/s13045-020-00972-5)
Supplement: Supplementary file 1 — Additional file 1: Table S1. The variant allele frequency of IKZF1 mutation in AML from cBioPortal database. [file 13045_2020_972_MOESM1_ESM.docx]

**Additional file 1: Table S1. The variant allele frequency of *IKZF1* mutation in AML from cBioPortal database.**

| **Study** | **Diagnosis** | **Mutation** | **VAF** |
| --- | --- | --- | --- |
| OHSU | AML with myelodysplasia-related changes | N159S | 11% |
|  | Therapy-related myeloid neoplasms | N159S | 29% |
|  | AML with myelodysplasia-related changes | N159S | 16% |
|  | Atypical chronic myeloid leukemia, *BCR-ABL-* | N159S | 28% |
|  | AML with myelodysplasia-related changes | Q44* | 15% |
|  | APL with *PML-RARA* | E80* | 28% |
|  | AML with t(3;3)(q21;q26.2); *GATA2, MECOM* | G323Wfs*166 | 21% |
|  | AML with myelodysplasia-related changes | E443Afs*21 | 15% |
| TCGA | AML | N130S | 40% |
| TARGET | AML | N159S | 33% |
|  | AML | R502W | 42% |
|  | AML | C175Y | 52% |
|  | AML | K380* | 42% |

**AML**, Acute myeloid leukemia; **APL**, acute promyelocytic leukemia; **VAF**, variant allele frequency.
